# On the Wrong Tack—The General Practitioners and Hospital Abuse

**Published:** 1906-10-20

**Authors:** 


### HOSPITAL ADMINISTRATION.

### CONSTRUCTION AND ECONOMICS.

### ON THE WRONG TACK—THE GENERAL PRACTITIONERS AND HOSPITAL ABUSE.

**The Sunday Fund and its Charter.**

The British Medical Association and the Charity Organisation Society have separately petitioned the Privy Council, and submitted for the Council's consideration certain points in relation to the charter of the Hospital Sunday Fund. They crave that His Majesty may be pleased to withhold his sanction to the grant of the proposed royal charter of incorporation to the Metropolitan Hospital Sunday Fund in its present form. It may be well to examine these points a little in detail. First of all the petition of the British Medical Association is submitted on behalf of the metropolitan counties branch, comprising 2,700 medical practitioners out of about 7,000 resident practitioners in London. It may be noted in passing that the petitioners number considerably less than one-half of the resident practitioners of the metropolis. The first point taken is that the Sunday Fund Council has the power to determine the conditions upon which contributions to hospitals should be granted, and so does exercise a controlling influence upon the administration of London hospitals. This point will not bear close examination, because the principle upon which the Sunday Fund has been conducted has been never to interfere in the internal management of any of the hospitals, for such interference might have destroyed the voluntary system. The object of the Sunday Fund is primarily the collection of money through the churches of all denominations upon one particular day in the year for the purpose of its systematic distribution through a Committee of Distribution representative of the medical profession and the public, but independent of the managers of the hospitals as such. The petition is therefore clearly inaccurate in stating that the Sunday Fund does exercise a controlling influence upon the administration of the London hospitals.

**The Medical Profession and the Hospitals.**

The petition next claims that the hospitals probably depend for their existence as much upon the gratuitous services of members of the medical profession as upon the monetary gifts of the charitable public. We have had occasion to point out more than once, however, that this contention is based upon a fallacy, for it would be good business for the hospitals to abolish gratuitous medical services, for then the authorities could enforce stricter economy, especially in relation to those departments, the expenditure upon which is capable of being largely reduced by a careful consideration of the relative cost of various articles ordered by the medical staff and used largely in the treatment of cases. The same contention applies with even greater force to the limitation of the number of patients admitted to treatment, a branch of the question which can never be adequately disposed of until the consultants attached to the hospitals agree to restrict the number of cases within strictly defined and relatively narrow limits, which cannot be determined or maintained without the hearty co-operation of the visiting medical staff. It is, therefore, inaccurate and misleading to set forth in the petition that the existence of the hospitals probably depends as much upon the gratuitous services of members of the medical profession as upon the monetary gifts of the charitable public. We agree with the petitioners, however, in their contention that all medical practitioners, even those not on the staff of any hospital, are deeply interested in the administration of hospitals. So deeply are the general practitioners interested in this question that unless something is done to effectively check the tendency of the public in ever-increasing numbers to obtain free medical relief when ill, there can be no doubt that the general practitioner as he exists to-day must certainly be starved out of existence. We make bold to say, however, that the general practitioners who, it is claimed, have presented this petition to the Privy Council are by this step merely beating the air. The Sunday Fund is practically powerless to effect the changes which are undoubtedly called for in regard to the ever-increasing volume of free medical relief granted by the hospitals. Hence the general practitioners should address themselves to the members of the visiting staffs of the great hospitals, where this free relief is at present given, for if the general practitioners combined in such a representation their voice must produce speedy and vital results, seeing that the visiting members of the staffs are largely dependent upon the great body of medical practitioners for most of the cases which they attend in their private consulting rooms. If the visiting staffs, moved by the general practitioners, were once to determine that the excess in the volume of free medical relief at present given by the hospitals must cease, the whole difficulty would end and the present abuses would disappear once and for all.

**Medical Practitioners on the Council and on the Committee of Distribution.**

The petitioners urge that the Sunday Fund fails to provide for any representation of medical practitioners upon these bodies. Here, again, the petitioners have fallen into error, for of fifty lay members on the Council ten are medical men of the highest standing in their profession, and of nine members of the Distribution Committee two are medical practitioners of eminence, namely, Sir William Church, Bart., and Mr. A. Willett, F.R.C.S., both St. Bartholomew's Hospital men, and so representing a non-participating hospital.

**Defects in our Hospital System.**

The petitioners, whilst admitting the beneficent work carried on by the Sunday Fund in many respects, declare themselves to be constrained to recognise that the Fund, as an almoner of public money, has failed to exercise the influence it might have done in removing serious defects in our hospital system. The defects pointed out are the evils of the out-patient department, the small payments exacted from out-patients at several hospitals, the failure to confine the out-patient department mainly to cases sent by general practitioners or provident dispensaries for consultation, the admission of trivial cases of accident and sudden illness to the casualty department, the failure to confine admissions to cases solely recommended by medical practitioners, the abolition of free dispensaries and of out-patient departments in favour of provident dispensaries, the non-reference of all destitute cases to the poor-law, and the undertaking of cases which are neither clinically nor financially suited for hospital treatment. Many of these questions have been the subject of discussion amongst the profession, the public, and the managers of hospitals for a generation at least. Their solution is primarily dependent upon the hearty co-operation of the visiting medical staffs of the hospitals, and with them must rest the first and most important step to remove the existing evils and so pave the way for the necessary reforms. The Hospital Sunday Fund Council has done its best to restrict abuse by its Distribution Committee laying down the rule that any hospital which has a special and efficient officer for detecting abuse shall have due credit given it on this ground when its merits are determined. How could this Fund, bearing in mind its policy of non-interference, better exercise such influence as it possesses to check the abuses complained of by the petitioners?

**Co-operation and Co-ordination of Hospitals and Other Medical Charities.**

The Charity Organisation's petition makes a great point of the importance of amalgamating the King's Fund with the Hospital Sunday Fund. Such an amalgamation must involve questions of principle and finance. In principle many people favour amalgamation, but there is much to be said against it, and we are confident the time is not ripe for such a step to be taken if the wisest and most matured counsel is to decide a point of this importance. Financially too amalgamation, whilst it might to an extent reduce the cost of management, though we rather doubt it, must inevitably entail risks with regard to the Sunday Fund and its collections, which in our view it is best to avoid. It is one thing to have a great Fund which offers a neutral platform whereon the representatives of all religious bodies can unite in the cause of the sick. It is quite another to merge this principle with many other objects which might introduce controversy and differences of opinion, and so tend to weaken the organisation by undermining the main plank on which the Sunday Fund must always rest, if the maximum of success is to be secured. We have never been able to see why the British Medical Association should have put itself in opposition to the Hospital Sunday Fund by petitioning against the grant of a royal charter. We believe this action to have been ill-considered and unwise. We are further sorry to note the narrowness of view which characterises much of the petition of the British Medical Association, especially the fifth section, which utterly ignores the claims and influence which rightly attach to (*a*) the needs of the self-respecting and deserving poor and their difficulties in securing prompt and adequate medical relief; (*b*) the almoners of the various associations who are constantly working amongst the poor and who have the best knowledge of their requirements and needs; and (*c*) the district nurses who must, under our modern system, if properly utilised, have a material influence for good in securing the removal of many abuses which at present attach to the ever-extending amount of free medical relief given by the London hospitals.

